# Oral Exposure to Low Concentration of Fumonisin B2, but Not Fumonisin B1, Significantly Exacerbates the Pathophysiology of Imiquimod-Induced Psoriasis in Mice

**DOI:** 10.3390/ijms25147852

**Published:** 2024-07-18

**Authors:** Mana Ando, Hiroki Yamaguchi, Naoki Iwashita, Yoshiichi Takagi, Tomoya Yoshinari, Tomoki Fukuyama

**Affiliations:** 1 Laboratory of Veterinary Pharmacology, School of Veterinary Medicine, Azabu University, 1-17-71 Fuchinobe, Chuo-ku, Sagamihara City 252-5201, Kanagawa, Japan; 2 Bioalch Co., Ltd., 3-28 Honshuku-cho, Fuchu City 183-0032, Tokyo, Japan; 3 Japan SLC, Inc., 85 Ohara-cho, Chuo-Ku, Hamamatsu City 431-1103, Shizuoka, Japan; 4 Division of Microbiology, National Institute of Health Sciences, 3-25-26 Tonomachi, Kawasaki-ku, Kawasaki City 210-9501, Kanagawa, Japan; 5 Center for Human and Animal Symbiosis Science, Azabu University, 1-17-71 Fuchinobe, Chuo-ku, Sagamihara City 252-5201, Kanagawa, Japan

**Keywords:** fumonisin B1, fumonisin B2, IL-17, IL-22, psoriasis

## Abstract

This study aimed to determine whether oral fumonisin exposure contributes to the development of psoriasis. Oral administration of fumonisin B1 (FB1, 0.1 mg/kg) or fumonisin B2 (FB2, 0.1 mg/kg) was conducted for 10 days, in addition to the induction of psoriatic symptoms through topical application of 5% imiquimod cream from day 6 to day 10 (5 days) in female BALB/c mice. The results demonstrated that oral administration of FB2 significantly exacerbated psoriatic symptoms, including skin thickness, itching behavior, transepidermal water loss, immune cell infiltration in the dermis, and proinflammatory cytokine production. However, no changes were observed following exposure to FB1. Our results confirm that oral exposure to FB2 adversely affects the pathogenesis of psoriasis by increasing skin thickness and impairing barrier function.

## 1. Introduction

The contamination of food and animal feed with mycotoxins, including aflatoxins, fumonisins, ochratoxins, zearalenone, and trichothecenes, is a global challenge [1,2]. Fumonisin, a mycotoxin produced by *Fusarium* spp., poses a major threat, with widespread distribution across the globe [3]. A recent epidemiological study detected fumonisin in 60% of 74,821 samples collected from 100 countries between 2008 and 2017, with the highest median concentration at 723 µg/kg [4]. Despite this high exposure risk, the detailed adverse effects of fumonisins, particularly on the immune system, remain poorly understood. Exposure to fumonisin reportedly causes encephalomalacia in horses, pulmonary edema in pigs, neural tube defects in human fetuses, and esophageal cancer in humans. These effects are potentially caused by fumonisin’s inhibition of the sphingolipid biosynthesis pathway [5,6,7]. The chemical structure of fumonisin resembles that of sphingolipids such as sphinganine and sphingosine. This similarity allows it to inhibit ceramide synthase through competitive antagonism, leading to the accumulation of and reduction in ceramide-related lipids [8]. Fumonisin B is a secondary metabolite produced by *Fusarium* spp., such as *Fusarium verticillioides* and *F. proliferatum.* Fumonisin B comprises fumonisin B1 (FB1) and B2 (FB2), both of which exhibit a markedly similar inhibitory pattern in ceramide synthase and are most frequently detected in foods [9,10,11].

Ceramide, produced from sphinganine through the ceramide synthesis pathway, is a major lipid component in the intercellular space of the stratum corneum. Recently, ceramide has been associated with the formation of the epidermal permeability barrier and several immune cells [12]. Disruption of the ceramide synthesis pathway has been linked to immunomodulatory diseases such as dermatitis, psoriasis, and inflammatory bowel disease [8,13,14]. We hypothesized that inhibition of ceramide synthase through oral exposure to FB1 or FB2 could accelerate the progression of these immunomodulatory diseases. As one of the prevalent immunomodulatory diseases, we focused on psoriasis, which is an autoimmune skin disease characterized by epidermal hyperplasia, leukocyte infiltration into the dermis and epidermis, and blood vessel dilation. An epidemiological study in 2013 (Parisi et al., 2013 [15]) indicated that the prevalence of psoriasis is 2–4% in the population of the USA and Europe, and the prevalence of psoriasis is increasing in Japan, potentially due to the westernization of the population’s diet (Yamashita et al., 2019 [16]). Therefore, the main objective of the present study was to determine and compare the adverse effects of oral FB1 and FB2 exposure on psoriasis using a suitable mouse model.

## 2. Results

### 2.1. Oral Exposure to FB2, but Not FB1, Significantly Exacerbated the Inflammatory Response in a Mouse Model of Imiquimod-Induced Psoriasis

**Figure 1:**
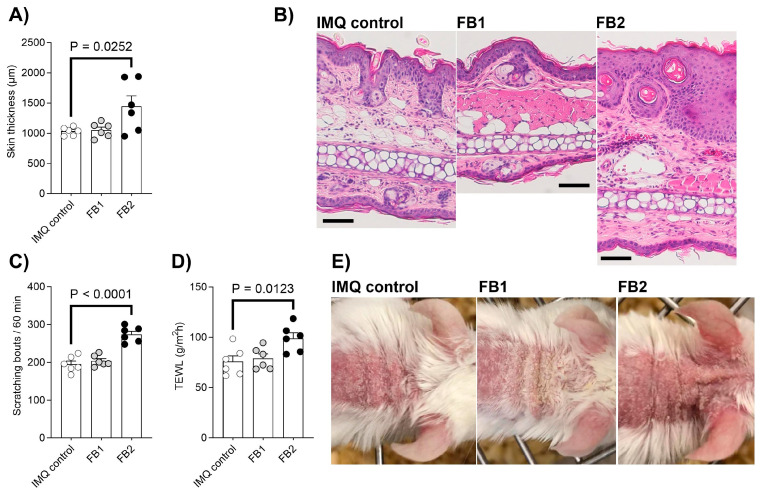
Effect of FB1 and FB2 exposure on an imiquimod (IMQ)-induced mouse model of psoriasis. IMQ-induced changes in back skin thickness (mm) (**A**), histology of auricular skin (scale bar =100 µm) (**B**), number of scratching bouts recorded in 60 min (**C**), transepidermal water loss (TEWL, g/m^2^/h) (**D**), and back skin appearance (**E**). All results are presented as the mean ± SEM and n = 6 per group. Statistical significance between the fumonisin-treated groups and the imiquimod control group was determined using Dunnett’s multiple comparisons test.

The present study investigated the in vivo interaction between oral exposure to FB1 or FB2 and the development of psoriatic lesions associated with the Th17 immune response using a mouse model of imiquimod-induced psoriasis. FB2 exposure significantly increased the inflammatory response, as evidenced by increased back skin thickness (Figure 1A). In contrast, FB1 exposure had no such effect. Histological analysis corroborated the FB2-induced increase in skin thickness, revealing significant inflammatory cell infiltration in the dermis of ear auricles (Figure 1B and Table 1). In contrast, FB1 exposure significantly reduced inflammatory cell infiltration in the epidermis of ear auricles compared to the imiquimod control group. Th17-dependent psoriasis is accompanied by a strong itching response. FB2 exposure significantly enhanced itching behavior compared to the imiquimod control group, whereas FB1 exposure had no impact (Figure 1C). FB2 exposure also resulted in a significant increase in transepidermal water loss (TEWL), a parameter of cutaneous barrier function, compared to the imiquimod control group (Figure 1D,E), while FB1 exposure resulted in no change.

### 2.2. Influence of FB1 or FB2 Exposure on Immune Function in Auricular Lymph Nodes in a Mouse Model of Psoriasis

**Figure 2:**
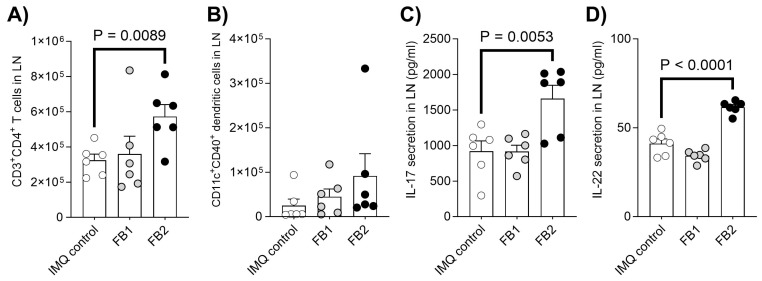
Influence of FB1 or FB2 exposure on immune function in auricular LN. Helper T cell (**A**) and dendritic cell (**B**) infiltration in the auricular lymph nodes (LNs) and IL-17 (**C**) and IL-22 (**D**) secretion (pg/mL) from auricular LNs were analyzed in IMQ-induced mice treated with FB1, FB2, or solvent alone. All results are presented as the mean ± SEM and n = 6 per group. Statistical significance between the fumonisin-treated groups and imiquimod control group was determined using Dunnett’s multiple comparisons test.

The effects of FB1 or FB2 exposure on immune function were investigated by analyzing the immune cell population and cytokine production in the auricular lymph nodes (LNs). Compared to the imiquimod control group, the number of helper T cells (CD3+ CD4+) was significantly elevated in the FB2-exposed group but remained unchanged in the FB1-exposed group (Figure 2A). Only a slight increasing trend was observed in the number of dendritic cells (CD11c+ MHC class II+) in both FB1- and FB2-exposed groups, but the changes were not statistically significant when compared to the imiquimod control group (Figure 2B). Th17-related immune function was assessed by measuring interleukin (IL)-17 and IL-22 production in CD3/CD28-stimulated T cells. FB2 exposure significantly enhanced IL-17 and IL-22 production compared to the imiquimod-exposed group (Figure 2C,D). However, no such effect was observed upon FB1 exposure.

## 3. Discussion

Psoriasis is an autoimmune skin disease characterized by epidermal hyperplasia, leukocyte infiltration into the dermis and epidermis, and blood vessel dilation. The exact cause of psoriasis is not fully understood, but recent evidence suggests that environmental factors, such as smoking, stress, obesity, and alcohol consumption, as well as a genetic predisposition to inflammation, may be involved in the development of psoriasis [17,18]. An epidemiological study in 2013 [15] indicated that the prevalence of psoriasis is 2–4% in the population of the USA and Europe, with a lower prevalence reported in Asian countries. However, the prevalence of psoriasis is increasing in Japan, potentially due to the westernization of the population’s diet [16]. Our study focused on the potential role of mycotoxin as an environmental factor in allergic diseases. Aihara et al. (2020) [19] and Ookawara et al. (2021) [20] demonstrated a relationship between oral exposure to low deoxynivalenol concentrations and allergies. Matsuzaka et al. (2024) [21] recently reported another trichothecene mycotoxin, nivalenol, and demonstrated that it directly activates mitogen-activated protein kinase signaling in antigen-presenting cells. Ando et al. (2023) [22] demonstrated that oral exposure to FB2 directly affects intestinal regulatory T cells, decreasing IL-10 secretion. This, in turn, exacerbated atopic dermatitis and allergic asthma in a mouse model. Since fumonisins exert their adverse effects by inhibiting the ceramide synthesis pathway, we aimed to investigate the influence of oral exposure to major fumonisins (FB1 and FB2) on the development of psoriasis, given its similarities with other inflammatory skin diseases.

In the present study, the interaction between fumonisin exposure and psoriasis development was investigated using an imiquimod-induced mouse model of psoriasis. This model effectively mimics human psoriasis, particularly the type associated with Th17 immune response modulation, and shares many pathogenetic, clinical, and histological features [23]. Our findings revealed that the ear-swelling response and epidermal thickness were significantly higher in the FB2-exposed group than in the control group. Psoriasis is characterized by excessive inflammation of the skin, impaired skin barrier function, and itching [24]. The significant increase in TEWL in the FB2-exposed group, but not the FB1-exposed group, indicates that FB2 exposure affects skin barrier function. As mentioned above, fumonisins exert their adverse effects by inhibiting the ceramide synthesis pathway. Riley, et al. [25] demonstrated that FB2 diets caused significant disruption of sphingolipid metabolism and caused hepatotoxicity and clinical signs indicative of the onset of equine leukoencephalomalacia. Gao, et al. [26] also indicated that oral exposure to FB1 directly affects the ceramide enzyme activity or levels of ceramide. FB2 is a structural analog of FB1 (R=OH for FB1 and R=H for FB2), and it seems that the inhibitory profile of ceramide synthesis is comparable between FB1 and FB2. Therefore, ceramide synthesis is not the only factor to account for the significant increase in TEWL in the FB2-exposed group. This compromised skin barrier and inflammatory skin reactions lead to strong itching or paresthesia, as evidenced by the significantly increased scratching frequency in FB2-treated mice compared to control mice. Interestingly, FB1 exposure did not exacerbate the symptoms of psoriasis, including inflammation, skin barrier dysfunction, or itching. This aligns with our previous research using a mouse model of atopic dermatitis and asthma, where FB1 exposure did not aggravate inflammatory symptoms [22].

Psoriasis development is initiated by dendritic cell activation, leading to IL-23 production. IL-23, in turn, promotes the differentiation of Th17 and Th22 cells, which plays an important role in the development of psoriatic symptoms [27]. Therefore, we investigated whether oral fumonisin exposure hyperactivates Th17 and Th22 immune responses. Our results indicated that helper T cell and T cell-dependent IL-17 and IL-22 production significantly increased in the FB2-treated mice. A previous study reported elevated IL-23 production in IL-10 knockout mice [28]. Notably, our previous study demonstrated that FB2 inhibits IL-10 production from intestinal regulatory T cells [22]. Taken together, these results suggest that the overproduction of IL-17 and IL-22 in FB2-treated mice might be directly or indirectly induced by the inhibition of IL-10 secretion from regulatory T cells.

In summary, our results confirm that oral exposure to FB2 adversely affects the pathogenesis of psoriasis by increasing skin thickness and impairing barrier function. The mechanism by which oral exposure to FB2 aggravates psoriasis appears to be an increase in IL-17 and IL-22 production in local LNs. Despite being less abundant in nature than FB1, FB2 remains active even at low concentrations. Additionally, the relationship between the FB2-induced inhibition of ceramide synthesis and psoriasis development was further investigated. Currently, analysis of ceramide enzyme activity or the levels of ceramide in local skin tissue after oral exposure to FB2 is being undertaken.

## 4. Materials and Methods

### 4.1. Animals and Chemicals

Six-week-old female BALB/c mice, provided by Japan SLC, Inc. (Shizuoka, Japan), were used to develop a mouse model of psoriasis. The mice were housed under a 12 h daily light cycle at a temperature of 22 ± 3 °C and a humidity of 50 ± 20%. Food and water were provided ad libitum. All experiments were conducted in accordance with the Animal Care and Use Program of Azabu University (Approval No. 1910097).

Imiquimod cream (5%) was purchased from MOCHIDA PHARMACEUTICAL Co., Ltd. (Tokyo, Japan). FB1 and FB2 were prepared according to a previously described method [22]. In the present study, FB1 or FB2 (diluted in distilled water) were administered orally to the mice at 0.1 mg/kg body weight (b.w.). The aim of this study is to evaluate the potential toxicity of FB1 or FB2 at a level equal to or lower than the current Non-Observed Adverse Effect Level (NOAEL). The current NOAEL is set as 0.21 mg/kg b.w. based on the results of a subacute toxicity study in rats; therefore, 0.1 mg/kg b.w. was selected for this study. In addition, FB1 and FB2 are occasionally detected at high levels (5–20 mg/kg) in foods intended for animal or human consumption. 

### 4.2. A Mouse Model of Imiquimod-Induced Psoriasis

**Figure 3:**
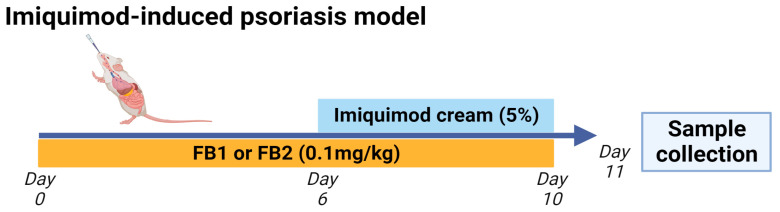
Experimental protocol used to sensitize and challenge the BALB/c mice with 5% imiquimod cream. The mice were orally administered either FB1 or fumonisin B2 (FB2) during the entire experimental period.

A mouse model of imiquimod-induced psoriasis was established by repetitive topical application of commercially available 5% imiquimod cream to the shaved rostral portion of the back and ear auricle for five consecutive days (days 6–10), as described in [29] (Figure 3). Briefly, the mice (n = 6 per group) received either 0.1 mg/kg b.w. of FB1 or FB2 orally once daily from days 0 to 10 (Figure 1). A control group (n = 6) received imiquimod cream without oral administration of FB1 or FB2. Scratching bouts (directed against the neck and ear regions) were monitored by video for 60 min after the final imiquimod cream application. To evaluate the inflammatory response, back skin thickness was measured 24 h after the final imiquimod cream application (cutimeter, Mitutoyo Corporation, Tokyo, Japan). To evaluate cutaneous barrier function, TEWL was simultaneously measured using a VAPO SCAN (AS-VT100RS, ASCH JAPAN Co., Ltd., Tokyo, Japan). Immediately after measuring skin thickness, the mice were euthanized by exsanguination from the abdominal aorta and posterior vena cava under isoflurane inhalation anesthesia. The ear auricle, back skin tissue, and auricular LNs were collected from each mouse and stored for further evaluation.

### 4.3. Histopathological Evaluation of the Ear Auricle and Back Skin

Portions of the ear auricle and back skin samples were fixed in 10% formalin solution, embedded in paraffin wax, sectioned to a thickness of 5 mm, and stained with hematoxylin and eosin. A pathologist blinded to the treatment groups performed the semi-quantitative histopathological evaluation of folliculitis, ulcers, edema, and cellular infiltration using the following grading system: 0, within normal limits; 1, mild; 2, moderate; 3, severe.

### 4.4. Flow Cytometric Analysis

Single-cell suspensions were obtained from the auricular LN using a previously described method [22]. The total number of cells was counted using a CellDrop™ Cell Counting system (DeNovix Inc., Wilmington, DE, USA). To avoid non-specific binding of antibodies during flow cytometric analysis, 1 × 10^6^ cells were first incubated with 1 µg of mouse Fc Block (Miltenyi Biotec K.K., Tokyo, Japan) prior to incubation with monoclonal antibodies (anti-mouse CD3, anti-mouse CD4, anti-mouse CD11c, and anti-mouse CD40 (BD Biosciences, Tokyo, Japan). The cells were also counterstained with 4′,6-diamidino-2-phenylindole (BD Biosciences, Tokyo, Japan). After incubation with monoclonal antibodies, the cells were then washed and analyzed using a BD FACSAria III cell sorter (BD Biosciences).

### 4.5. Cytokine Release Assay

Single-cell suspensions of LNs were also used to evaluate cytokine release by T cells. Single-cell suspensions of LNs (5 × 10^5^ cells/well) were incubated with Dynabeads mouse T-Activator CD3/CD28 (Thermo Fisher Scientific, Inc., Yokohama, Kanagawa, Japan) for 24 h. The concentrations of IL-17 and IL-22 (R&D Systems, Minneapolis, MN, USA) in the supernatant were evaluated using enzyme-linked immunosorbent assay. The optical density at 450 nm was measured using an iMark microplate reader (Bio-Rad Laboratories, Inc., Hercules, CA, USA).

### 4.6. Statistical Analysis

The statistical significance of the difference between the control and treatment groups was assessed at the 5% and 1% probability levels. Data from the control and treatment groups were evaluated using Bartlett’s test for homogeneity of variance. When the group variances were homogeneous, a parametric one-way analysis of variance was conducted to determine statistical differences among the groups. Dunnett’s multiple comparisons test was performed when the analysis of variance was significant. When the group variances were heterogeneous, the data were evaluated using the non-parametric Kruskal–Wallis analysis of variance, followed by Dunnett’s rank-sum test when significant differences were detected. All data are expressed as the mean ± standard error of the mean and were analyzed using Prism 10 software (GraphPad Software, San Diego, CA, USA).

## Figures and Tables

**Table 1:** Histological evaluation of the skin following oral administration of FB1 and FB2 in an imiquimod-induced mouse model of psoriasis.

Group	Imiquimod Control	FB1	FB2
Ear auricle			
Inflammatory cell infiltration in the epidermis	1.17 ± 0.17	0.33 ± 0.21 *	1.67 ± 0.21
Hyperplasia in the epidermis (keratinized layer)	0.33 ± 0.21	0.33 ± 0.21	0.33 ± 0.21
Hyperplasia in the epidermis (non-keratinized layer)	2.50 ± 0.22	2.50 ± 0.34	3.00 ± 0.00
Inflammatory cell infiltration in the dermis	1.33 ± 0.21	0.83 ± 0.17	2.83 ± 0.17 **
Back skin			
Inflammatory cell infiltration in the epidermis	1.50 ± 0.22	1.67 ± 0.21	2.00 ± 0.00
Hyperplasia in the epidermis (keratinized layer)	1.50 ± 0.34	2.00 ± 0.00	2.00 ± 0.00
Hyperplasia in the epidermis (non-keratinized layer)	3.00 ± 0.00	3.00 ± 0.00	3.00 ± 0.00
inflammatory cell infiltration in the dermis	2.83 ± 0.17	3.00 ± 0.00	3.00 ± 0.00

A histological score (0, within normal limits; 1, mild; 2, moderate; 3, severe) was given for each observation. The results are expressed as the mean ± SEM. n = 6 per group. * *p* < 0.05; ** *p* < 0.01. Statistical significance between the fumonisin-treated groups and the imiquimod control group was determined using Dunnett’s multiple comparisons test.

## Data Availability

The raw data supporting the conclusions of this article will be made available by the authors upon request.

## References

[1] Danicke S., Carlson L., Heymann A.K., Grumpel-Schluter A., Doupovec B., Schatzmayr D., Streit B., Kersten S., Kluess J. (2023). Inactivation of zearalenone (ZEN) and deoxynivalenol (DON) in complete feed for weaned piglets: Efficacy of ZEN hydrolase ZenA and of sodium metabisulfite (SBS) as feed additives. Mycotoxin Res..

[2] Munoz-Solano B., Gonzalez-Penas E. (2023). Co-Occurrence of Mycotoxins in Feed for Cattle, Pigs, Poultry, and Sheep in Navarra, a Region of Northern Spain. Toxins.

[3] Santiago R., Cao A., Malvar R.A., Butron A. (2020). Genomics of Maize Resistance to Fusarium Ear Rot and Fumonisin Contamination. Toxins.

[4] Gruber-Dorninger C., Jenkins T., Schatzmayr G. (2019). Global Mycotoxin Occurrence in Feed: A Ten-Year Survey. Toxins.

[5] Missmer S.A., Suarez L., Felkner M., Wang E., Merrill A.H., Rothman K.J., Hendricks K.A. (2006). Exposure to fumonisins and the occurrence of neural tube defects along the Texas-Mexico border. Environ. Health Perspect..

[6] Gordon R.J., Hungerford N.L., Laycock B., Fletcher M.T. (2020). A review on Pimelea poisoning of livestock. Toxicon.

[7] Alizadeh A.M., Rohandel G., Roudbarmohammadi S., Roudbary M., Sohanaki H., Ghiasian S.A., Taherkhani A., Semnani S., Aghasi M. (2012). Fumonisin B1 contamination of cereals and risk of esophageal cancer in a high risk area in northeastern Iran. Asian Pac. J. Cancer Prev..

[8] Wang E., Norred W.P., Bacon C.W., Riley R.T., Merrill A.H. (1991). Inhibition of sphingolipid biosynthesis by fumonisins. Implications for diseases associated with Fusarium moniliforme. J. Biol. Chem..

[9] Norred W.P., Plattner R.D., Dombrink-Kurtzman M.A., Meredith F.I., Riley R.T. (1997). Mycotoxin-induced elevation of free sphingoid bases in precision-cut rat liver slices: Specificity of the response and structure-activity relationships. Toxicol. Appl. Pharmacol..

[10] Rheeder J.P., Marasas W.F., Vismer H.F. (2002). Production of fumonisin analogs by Fusarium species. Appl. Environ. Microbiol..

[11] World Health Organization (2017). Evaluation of Certain Contaminants in Food.

[12] Sofi M.H., Tian L., Schutt S., Khan I., Choi H.J., Wu Y., Bastian D., Ticer T., Kassir M.F., Atilgan F.C. (2022). Ceramide synthase 6 impacts T-cell allogeneic response and graft-versus-host disease through regulating N-RAS/ERK pathway. Leukemia.

[13] Komuro M., Nagane M., Fukuyama T., Luo X., Hiraki S., Miyanabe M., Ishikawa M., Niwa C., Murakami H., Okamoto M. (2022). Sphingomyelin maintains the cutaneous barrier via regulation of the STAT3 pathway. FASEB J..

[14] Komuro M., Nagane M., Endo R., Nakamura T., Miyamoto T., Niwa C., Fukuyama T., Harashima H., Aihara N., Kamiie J. (2022). Glucosylceramide in T cells regulates the pathology of inflammatory bowel disease. Biochem. Biophys. Res. Commun..

[15] Parisi R., Symmons D.P., Griffiths C.E., Ashcroft D.M., on behalf of the Identification and Management of Psoriasis and Associated ComorbidiTy (IMPACT) Project Team (2013). Global epidemiology of psoriasis: A systematic review of incidence and prevalence. J. Investig. Dermatol..

[16] Yamashita H., Morita T., Ito M., Okazaki S., Koto M., Ichikawa Y., Takayama R., Hoashi T., Saeki H., Kanda N. (2019). Dietary habits in Japanese patients with psoriasis and psoriatic arthritis: Low intake of meat in psoriasis and high intake of vitamin A in psoriatic arthritis. J. Dermatol..

[17] Hu M., Wang Y., Xu W., Bai J., Tang X. (2024). The impact of serum uric acid on psoriasis: NHANES 2005–2014 and Mendelian randomization. Front. Genet..

[18] Gaurav V., Anand G.R.P., Grover C. (2024). Follicular Psoriasis: A Case Report and Review of Literature. Ski. Appendage Disord..

[19] Aihara R., Ookawara T., Morimoto A., Iwashita N., Takagi Y., Miyasaka A., Kushiro M., Miyake S., Fukuyama T. (2020). Acute and subacute oral administration of mycotoxin deoxynivalenol exacerbates the pro-inflammatory and pro-pruritic responses in a mouse model of allergic dermatitis. Arch. Toxicol..

[20] Ookawara T., Aihara R., Morimoto A., Iwashita N., Kurata K., Takagi Y., Miyasaka A., Kushiro M., Miyake S., Fukuyama T. (2021). Acute and Subacute Oral Toxicity of Deoxynivalenol Exposure in a Dermatophagoides fari-nae-Induced Murine Asthma Model. Toxicol. Sci..

[21] Matsuzaka R., Yamaguchi H., Ohira C., Kurita T., Iwashita N., Takagi Y., Nishino T., Noda K., Sugita K., Kushiro M. (2024). Sub-acute oral exposure to lowest observed adverse effect level of nivalenol exacerbates atopic dermatitis in mice via direct activation of mitogen-activated protein kinase signal in antigen-presenting cells. Arch. Toxicol..

[22] Ando M., Yamaguchi H., Morimoto A., Iwashita N., Takagi Y., Nagane M., Yoshinari T., Fukuyama T. (2023). Chronic oral exposure to low-concentration fumonisin B2 significantly exacerbates the inflammatory responses of allergies in mice via inhibition of IL-10 release by regulatory T cells in gut-associated lymphoid tissue. Arch. Toxicol..

[23] Van der Fits L., Mourits S., Voerman J.S., Kant M., Boon L., Laman J.D., Cornelissen F., Mus A.M., Florencia E., Prens E.P. (2009). Imiquimod-induced psoriasis-like skin inflammation in mice is mediated via the IL-23/IL-17 axis. J. Immunol..

[24] Chen H., Wang C., Tang B., Yu J., Lu Y., Zhang J., Yan Y., Deng H., Han L., Li S. (2021). *P. granatum* Peel Polysaccharides Ameliorate Imiquimod-Induced Psoriasis-Like Dermatitis in Mice via Suppression of NF-kappaB and STAT3 Pathways. Front. Pharmacol..

[25] Riley R.T., Showker J.L., Owens D.L., Ross P.F. (1997). Disruption of sphingolipid metabolism and induction of equine leukoencephalomalacia by Fusarium proliferatum culture material containing fumonisin B(2) or B(3). Environ. Toxicol. Pharmacol..

[26] Gao Z., Luo K., Zhu Q., Peng J., Liu C., Wang X., Li S., Zhang H. (2023). The natural occurrence, toxicity mechanisms and management strategies of Fumonisin B1: A review. Environ. Pollut..

[27] Kagami S. (2011). IL-23 and Th17 cells in infections and psoriasis. Nihon Rinsho Meneki Gakkai Kaishi.

[28] Kamada N., Hisamatsu T., Okamoto S., Sato T., Matsuoka K., Arai K., Nakai T., Hasegawa A., Inoue N., Watanabe N. (2005). Abnormally differentiated subsets of intestinal macrophage play a key role in Th1-dominant chronic colitis through excess production of IL-12 and IL-23 in response to bacteria. J. Immunol..

[29] Yamaguchi H., Ando M., Iwashita N., Takagi Y., Kushiro M., Fukuyama T. (2023). Oral exposure to citrinin significantly exacerbates the pathophysiology of a mouse model of imiquimod-induced psoriasis via direct activation of dendritic cell. J. Appl. Toxicol..

